# Epidemiological, clinical and laboratory characteristics of the measles resurgence in the Republic of Serbia in 2014-2015

**DOI:** 10.1371/journal.pone.0224009

**Published:** 2019-10-17

**Authors:** Snežana Medić, Vladimir Petrović, Goranka Lončarević, Milena Kanazir, Ivana Begović Lazarević, Slavica Rakić Adrović, Maja Bančević, Claude P. Muller, Judith M. Hübschen

**Affiliations:** 1 Center for Disease Control and Prevention, Institute of Public Health of Vojvodina, Novi Sad, Serbia; 2 Faculty of Medicine, University of Novi Sad, Novi Sad, Serbia; 3 Department for Disease Control and Prevention, Institute of Public Health of Serbia, Belgrade, Serbia; 4 Center for Disease Control and Prevention, Institute of Public Health of Belgrade, Belgrade, Serbia; 5 Institute of Laboratory Medicine, SLK Clinics, Heilbronn, Germany; 6 National Reference Laboratory for Measles and Rubella, Institute of Virology, Vaccine and Sera ‘Torlak’, Belgrade, Serbia; 7 WHO European Regional Reference Laboratory for Measles and Rubella, Department of Infection and Immunity, Luxembourg Institute of Health, Esch-sur-Alzette and Laboratoire National de Santé, Dudelange, Luxembourg; University of New South Wales, AUSTRALIA

## Abstract

The Republic of Serbia is a country with ongoing endemic transmission of measles. The aim of this study is to summarize the main characteristics of the measles resurgence that occurred in Serbia in 2014–2015. The national surveillance data on measles was analysed in relation to the clinical, epidemiological and laboratory data. Between November 2014 and December 2015 a measles resurgence with 420 cases was observed in Serbia. Measles virus was initially introduced by and spread among citizens of Bosnia and Herzegovina with temporary residence in Serbia, before spreading to the resident population. Of the 223 patients with available medical records, 173 (77.6%) were unvaccinated. The overall measles incidence during the outbreak was 5.8/100.000. The highest age-specific incidence rate was recorded in children aged ≤4 years (25.9/100.000), but most cases (67.9%) were ≥20 years old. Hospitalization rate was high (32.9%) and included two cases of encephalitis associated with measles. In total, 42 health-care workers and 22 related cases including hospitalized patients (n = 13) contracted measles. The overall percentage of laboratory confirmed cases was 81.7% (n = 343/420). All measles virus sequences except one (D9) belonged to genotype D8, suggesting interruption of transmission after the previous outbreak in 2010–2011 caused by genotype D4 viruses. The growing number of adult patients as compared to previous epidemics, suggests an urgent need for supplementary immunization activities targeting susceptible health care workers, unvaccinated or incompletely vaccinated adults as well as people without vaccination records. The comprehensive investigation of the 2014/2015 measles resurgence will contribute to decisions about appropriate countermeasures to stop the future measles resurgences in Serbia.

## Introduction

Despite the availability of vaccine for more than 50 years, measles still remains one of the leading causes of global child mortality [1,2]. Elimination of measles in the European Region of the World Health Organization (WHO) targeted for 2015 was not achieved [3,4], leading to the adoption of the Global Measles and Rubella Strategic Plan, 2012–2020 and The European Vaccine Action Plan 2015–2020 [5,6]. Over 30000 cases of measles were registered in Europe in 2015, including including 368 in Serbia [7].

The Republic of Serbia (Serbia) is a country located in Southeast Europe with a population of about 7 million inhabitants and a birth cohort size of about 70 000 [8]. Surveillance based on the WHO measles case definition was implemented in 2009 [9] and relies on the 22 Institutes of Public Health (IPH)-governmental organizations involved in communicable disease prevention and control, the National Reference Laboratory for measles (NRL) and health-care workers (HCWs) at all levels of health care. Mandatory, free-of-charge vaccination against measles was introduced in 1971, given as a single dose of monovalent vaccine at the age of 12–15 months. In 1986, a single dose of combined measles-mumps (MM) vaccine was introduced. As of 1993, measles-mumps-rubella (MMR) vaccine replaced the MM vaccine. A two-dose MMR schedule was introduced in 1996 with the second dose given at 12 years of age. Since 2006, the second dose is scheduled at the age of seven years [10].

Between 2001 and 2011, vaccination coverage for the first dose of MMR was continuously above 95%, while the coverage for the second dose ranged from 84% to 98% [11]. In the period 2012–2015, coverage dropped to <95% for both doses due to frequent vaccine shortages and anti-vaccination movements [11, 12]. Between 2000 and 2006, measles incidence was below 0.5/100.000 inhabitants [11]. In 2007, an outbreak of measles in the Autonomous Province of Vojvodina (Vojvodina) in the North of the country affected mostly unvaccinated Roma children between 1 and 14 years of age [13]. Until 2010 only sporadic cases were recorded. In 2010 and 2011, a measles outbreak occurred in Southeast Serbia and affected at least 363 people, mostly unvaccinated children up to 4 years of age [14]. After 2011, only two sporadic measles cases were recorded until end of 2014, when yet another resurgence of measles was observed. This manuscript summarizes the main characteristics of the measles resurgence in Serbia in 2014–2015, identifies its causes and discusses interventions to prevent future epidemics.

## Materials and methods

### Ethics statement

The investigation of the measles resurgence in Serbia was done in the frame of national public health surveillance. Sample collection for laboratory diagnosis was part of standard patient management and required only oral informed patient consent. Data on suspected measles cases were reported by the physicians to the IPH on a daily basis. Access to patient data was restricted to employees directly involved in measles diagnosis and reporting.

### Case definition and classification

A suspected measles case is defined as a patient with fever and a maculopapular rash and at least one of the following three symptoms: cough, coryza or conjunctivitis. Final classifications of measles cases as clinically compatible, epidemiologically linked or laboratory confirmed cases as well as definition by origin (endemic, imported, import-related, unknown) were done according to the WHO criteria [15]. Two or more laboratory confirmed cases which are temporally related and epidemiologically and/or virologically linked, were considered as an outbreak [15].

### Data sources

Epidemiological data were obtained from the surveillance database of the IPH of Serbia, Belgrade and the IPH of Vojvodina, Novi Sad. Data originate from the mandatory notification forms, epidemiological questionnaires and medical records and include demographic data including occupation, residence and ethnicity, date of symptom onset, clinical characteristics, hospitalization status, case confirmation method, measles vaccination status, travel and contact history, potential epidemiological links and disease outcome. Final case classifications were done by the IPH of Serbia, after completion of the laboratory and epidemiological investigations.

### Sample collection, laboratory testing and phylogenetic analysis

Clinical samples were normally collected by trained laboratory technicians upon request of an epidemiologist as part of routine, case-based surveillance. The epidemiologist also decided which specimens were taken, based on a case evaluation and according the algorithm for specimen collection [9]. A large number of suspected cases required the limitation of laboratory testing to cases from new locations and without epidemiological links to laboratory-confirmed cases. Due to the various reasons such as refusal and unavailability of follow-up, samples could not be collected from all suspected cases without an epidemiological link. Sera for the detection of measles-specific IgM antibodies were collected within 3–28 days after rash onset, while nose/throat swabs for detection of viral RNA were obtained within seven days of rash onset. In the laboratories of the IPHs, different commercial ELISA kits were used for IgM antibody testing and only the NRL performed viral RNA detection by PCR, as described before [14]. In total, 420 sera (including two follow up sera from one patient) and 213 nose/throat swabs were collected between November 2014 and December 2015.

To assess whether most patients had primary contact with measles virus, IgG avidity was checked for 57 IgM positive sera using a commercial ELISA kit (Euroimmun, Lübeck, Germany). Serum samples were conveniently selected for avidity testing with a similar distribution in respect to age, sex, and vaccination status as the total laboratory confirmed sera specimens. The samples had been collected from 28 females and 29 males between 1 and 54 years of age. Thirteen patients were unvaccinated, 38 had an unknown vaccination status, and 6 patients were vaccinated, 4 of them with a single dose and 2 with two doses of MMR. All PCR positive nose/throat swabs were used to attempt measles virus genotyping.

The swabs had been collected from patients aged ≤1 year up to 50 years of age. Initial sequence analysis was done using SeqScape Software v2.5 (Applied Biosystems, USA), followed by BioEdit version 7.0.9.0 [16]. Phylogenetic trees were generated based on the Kimura 2-parameter model, the Neighbor-Joining algorithm and 450 nucleotides (nt) of the measles virus nucleoprotein using MEGA6 software [17]. The most recent reference sequences [18], all named strains of genotype D8 and some BLAST (basic local alignment search tool that finds regions of similarity between biological sequences) fits of Serbian sequences were included in the analysis. The sequences from Serbia were named according to WHO nomenclature with location as well as rash onset date by epidemiological week and year [19] and are available on MeaNS [20] and on GenBank under accession numbers MH249631-MH249767.

## Results

### Descriptive epidemiology

**Fig 1:**
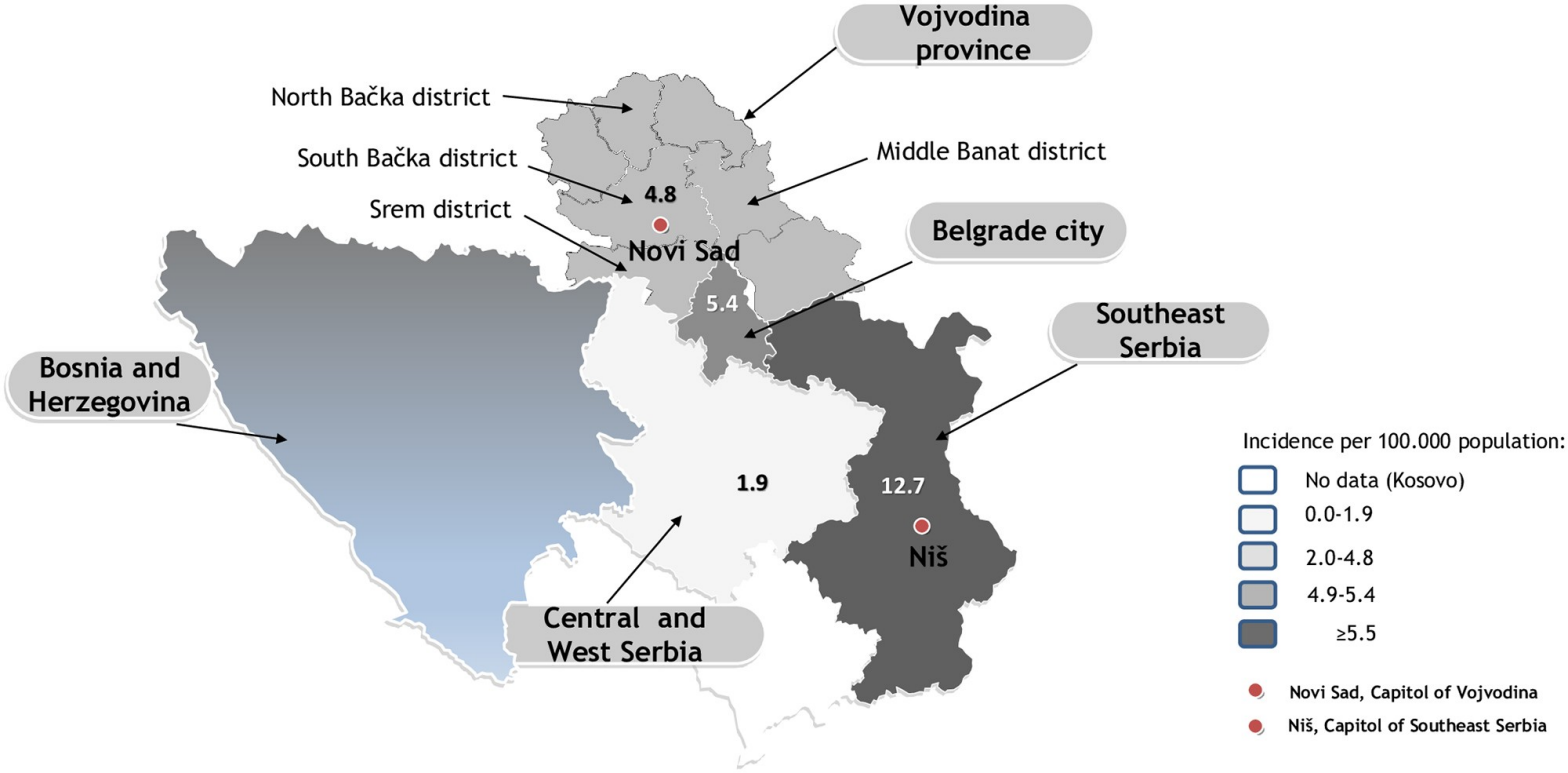
Incidence rates of measles per 100 000 inhabitants in the four major administrative regions in Serbia, 2014–2015. Measles outbreaks occurred in four regions of Serbia (Vojvodina province, Belgrade city, Central and West Serbia and Southeast Serbia).

Between November 2014 and December 2015, a resurgence of measles was registered in Serbia. The first cases occurred in the northern province Vojvodina (Fig 1).

**Fig 2:**
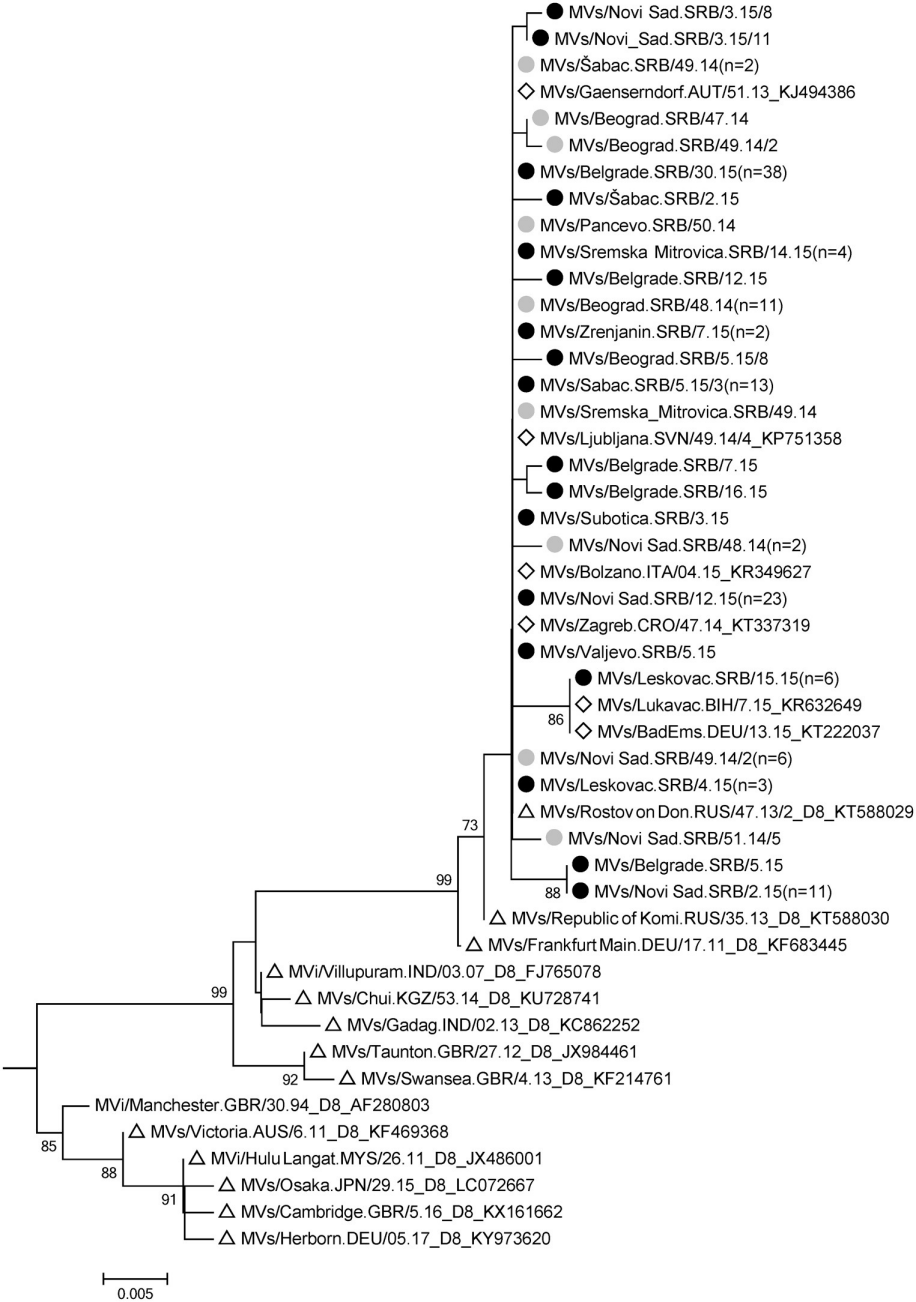
D8 cluster of a phylogenetic tree constructed based on 450 nucleotides of the measles virus N gene using the Kimura 2-parameter model and the Neighbor-Joining algorithm. The Serbian sequences are marked with grey dots if they were from 2014 and with black dots if observed in 2015. For the 2014 sequences the strain detected at the earliest time point was selected, for 2015 the one found at the latest time point. Numbers in brackets indicate identical sequences from the same year and location. Triangles mark genotype D8 named strains with GenBank accession numbers and diamonds highlight identical BLAST fits of Serbian sequences.

The index case was a student of the University of Novi Sad, originating from Bileća, Republic of Srpska, Bosnia and Herzegovina (B&H), where he was in contact with a family member with measles. The first notified and laboratory confirmed case was another student, who had contact with the index case in Novi Sad, eight days before onset of symptoms (MVs/Novi Sad.SRB/48.14, Fig 2).

The outbreak originating from this case affected mostly student residences in South Bačka district and comprised 64 measles cases until end of March 2015. A one-and-a-half-year-old unvaccinated Roma child was the first case of measles in the Srem district, Vojvodina (MVs/Sremska_Mitrovica.SRB/49.14, Fig 2). According to the epidemiological data, he lived with his family in a home for asylum seekers in Berlin, Germany, in November 2014, where he was exposed to asylum seekers with measles. Seven days after returning to Serbia, the child experienced measles symptoms, but there were no secondary cases. Between January and April 2015, two districts of Vojvodina experienced outbreaks (North Bačka and Middle Banat) with overall 18 patients, mainly students who travelled to B&H (e.g. MVs/Subotica.SRB/3.15, Fig 2). Between February and April 2015 measles cases accumulated in the resident population of Vojvodina (e.g. MVs/Novi Sad.SRB/12.15, MVs/Zrenjanin.SRB/7.15, MVs/Sremska Mitrovica.SRB/14.15, Fig 2) without confirmed epidemiological link to the students. Overall 93 cases (4.8/100 000 inhabitants) were notified in Vojvodina until the end of the outbreak (April 2015). Nearly half of the patients (n = 41, 44.1%) were citizens of B&H with temporary residence in Vojvodina.

Between November 2014 and April 2015, a measles resurgence with 90 registered cases took place in Belgrade city. The first case was identified during the investigation of an epidemiologically related case (MVs/Beograd.SRB/46.14, genotype D9, Fig 2) and had been travelling through Asia for three consecutive weeks before falling ill on the last day of the trip. Two other measles cases, both of them citizens of B&H, were considered as imported from B&H based on epidemiological data. These cases caused import-related cases (e.g. MVs/Beograd.SRB/47.14, Fig 2) and according to epidemiological data were the probable source of the majority of subsequent measles cases in the outbreak in Belgrade. Another measles outbreak in Belgrade with three hospital-acquired cases lasted from June 18^th^ to July 21^th^ 2015. The index case (MVs/Belgrade.SRB/26.15, identical to “Rostov on Don” variant shown in Fig 2) was a two-year-old child, who had been hospitalized at the Niš Children’s Clinic (Southeast Serbia) seven days before admission to the hospital in Belgrade. This case was the source of infection for two mothers staying with their children in the same hospital (e.g. MVs/Belgrade.SRB/30.15, Fig 2).

An outbreak of measles in Southeast Serbia with 199 cases affected primarily Nišava district (86% of all cases) and spread among residents within schools, kindergartens, companies and healthcare institutions. For the cases occurring in a Roma settlement (e.g. MVs/Leskovac.SRB/15.15, Fig 2), a probable epidemiological link to Germany was identified. A total of 25 measles cases, including six hospitalized patients and 12 health-care workers (HCWs), were registered during an outbreak at the Niš Children’s Clinic between March 30 and June 10, 2015.

**Fig 3:**
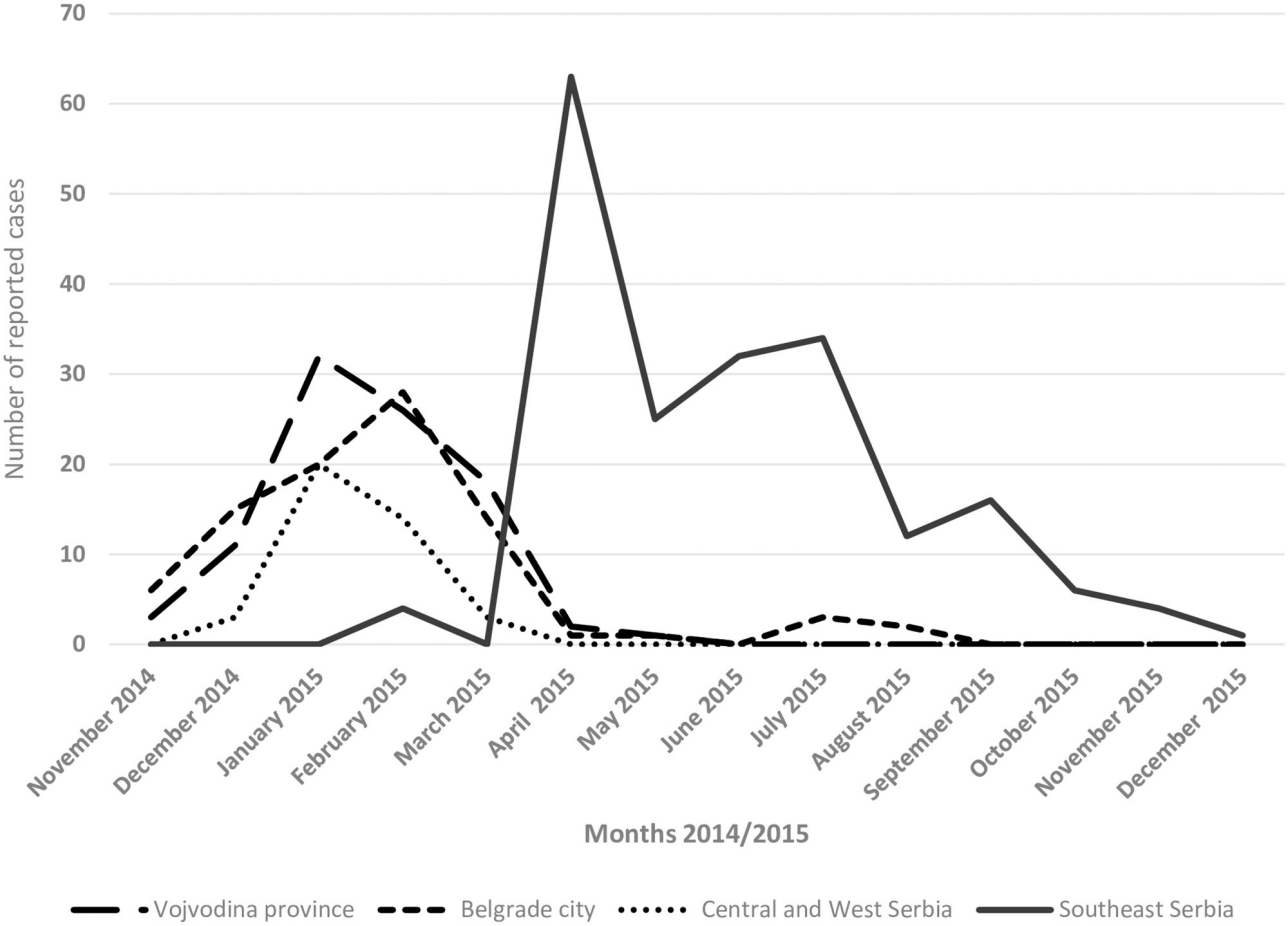
Number of measles cases by month of notification and residence in Serbia, 2014–2015.

Overall, 420 (67.3%) out of 624 suspected cases were classified as measles. In 2014, 37 measles cases were notified in Serbia, resulting in an incidence of 0.5/100 000 inhabitants. The rest of the cases occurred in 2015 (n = 383; incidence 5.4/100 000). The highest incidence was registered in Southeast (12.7/100 000) and the lowest in Central and West Serbia (1.9/100 000 citizens) (Fig 1). The overall measles incidence during the outbreak period was 5.8/100 000 population. The peak in the number of measles cases was observed in January and February 2015 in Vojvodina, Belgrade city, Central and West Serbia and in April 2015 in Southeast Serbia (Fig 3). The measles resurgence ended in December 2015 and no cases occurred until October 2016.

Eleven measles cases were classified as imported, most of them (n = 9) from Republic of Srpska, B&H. All of these 11 cases were laboratory confirmed and for eight of them sequence data were obtained (MVs/Novi Sad.SRB/49.14/2, MVs/Novi_Sad.SRB/2.15, MVs/Sremska_Mitrovica.SRB/49.14, MVs/Sabac.SRB/49.14, Fig 2; MVs/Novi Sad.SRB/49.14/3, MVs/Novi Sad.SRB/51.14/2, MVs/Beograd.SRB/49.14, identical to “Rostov on Don” variant shown in Fig 2; MVs/Novi_Sad.SRB/3.15/10, identical to MVs/Novi_Sad.SRB/2.15 shown in Fig 2). These cases have resulted in at least 22 import-related cases. For the majority of cases in this resurgence (n = 377, 89.8%) no source of infection could be established and they were considered as endemic. Data for the classification of ten cases were lacking, resulting in an unknown importation status (Table 1).

**Table 1:** Number of measles cases by case confirmation method and origin of infection in Serbia, 2014–2015.

Measles cases	Laboratory-confirmed	Epidemiologically linked	Total
**Imported**	11	0	11
**Import-related**	22	0	22
**Endemic**	302	75	377
**Unknown**	8	2	10
**Total**	343	77	420

Overall 42 employees of 17 healthcare institutions (12 physicians, 20 nurses, two lab workers, two medical students and six technical-supportive employees) became infected with measles. Six HCWs with measles were born before 1971 and therefore not vaccinated. A total of 28 HCWs were born between 1971 and 1984 (they should have received one dose of vaccine) of which eight had documented vaccination, two were unvaccinated, and the rest had an unknown vaccination status. Eight HCWs were born after 1984, but only two of them were fully vaccinated, one was unvaccinated and the remaining patients had an unknown vaccination status. The majority of infected HCWs acquired measles while working in a hospital (n = 29). At least 22 secondary cases occurred, most of them (n = 13) among hospitalized patients.

### Characteristics of patients

Similar numbers of males and females (51.3% *vs* 48.7%) were affected. The youngest patient was two months and the oldest 56 years old (median 28 years). Most patients were adults ≥20 years (n = 285; 67.9%). Almost one third of the cases (n = 124; 29.5%) were children aged 0–14 years. The age-specific incidence rates ranged from 2.5 (age 15–19 years) to 25.9 (age 0–4 years) per 100 000 population (Table 2)

**Table 2:** Age specific incidence and number of measles cases in Serbia 2014–2015 by age group and vaccination status.

Doses of measles containing vaccine	Age groups						
	0–4 n(%)	5–9 n(%)	10–14 n(%)	15–19 n(%)	20–29 n(%)	≥30 n(%)	Total n(%)
**0 dose**	80(93.0)	26(96.3)	5(45.5)	5(45.5)	30(38.0)	27(13.1)	173(41.2)
**1 dose**	6(7.0)	1(3.7)	6(54.5)	4(36.4)	2(2.5)	19(9.2)	38(9.0)
**2 or more doses**	0(0.0)	0(0.0)	0(0.0)	0(0.0)	7(8.9)	5(2.4)	12(2.9)
**Unknown**	0(0.0)	0(0.0)	0(0.0)	2(18.1)	40(50.6)	155(75.3)	197(46.9)
**Total number of cases**	**86(100.0)**	**27(100.0)**	**11(100.0)**	**11(100.0)**	**79(100.0)**	**206(100.0)**	**420(100.0)**
**Age specific incidence**^a^	**25.9**	**8.6**	**3.2**	**2.5**	**8.9**	**4.2**	**5.8**

^a^per 100.000 population

For more than half of the patients (n = 223; 53.1%) medical records were available showing that 5.4% had been vaccinated with two doses and 17.0% with one dose of measles virus-containing vaccine. Of the 77.6% (n = 173/223) unvaccinated patients, many were children ≤4 years of age (n = 80; 46.2%) and adults ≥20 years of age (n = 57; 32.9%). Patients with unknown vaccination status (n = 197/420; 46.9%) were mostly adults (99%)(Table 2). At least 80 Roma were affected including 58 children aged ≤14 years (70% unvaccinated and 27.6% with unknown vaccination status). The majority (93.7%) of Roma patients were residents of Southeast Serbia.

A total of 138 patients (32.9%) were hospitalized. The reasons were measles-related complications (30.4% of hospitalized patients) or as a precautionary measure because of severe disease. Excluding diarrhoea (n = 40, 29.0%), and malnutrition (n = 7; 5.1%), serious complications were reported in at least 42 hospitalized patients including pneumonia (n = 37; 26.8%), pleural effusion (n = 2; 1.4%) and erosion of the cornea (n = 1; 0.7%). Two cases of encephalitis associated with measles were registered, but there were no fatalities. Complications were mostly notified in patients above 30 years and below 4 years of age (45.2 and 23.8% respectively). The majority of them were unvaccinated (n = 20; 47.6%) or with unknown vaccination status (n = 19; 45.2%) and only three adult patients were vaccinated with one dose of vaccine more than 30 years ago (range 33–37 years).

### Laboratory findings

Out of 624 suspected measles cases, 526 (84.3%) were tested and 343 of them (65.2%) were laboratory confirmed, 198 by serology, 80 by PCR and 65 by both methods (Table 3).

**Table 3:** Laboratory results during the measles resurgence in Serbia, 2014–2015.

Sample	No. of patients	Serum IgM			Nose-throat swab (PCR)	
		Positive	Negative	Equivocal	Positive	Negative
Swab	106	/	/	/	80	26
Serum and swab	107	65	40	2	65	42
Serum	313	198	96	19	/	/
**Total**	**526**	**263**	**136**	**21**	**145**	**68**

A total of 37 sera showed low avidity, suggesting a recent primary infection, while the remaining 20 sera had high avidity measles IgG antibodies, indicating previous contact with measles wild-type or vaccine virus. All patients with low avidity were either unvaccinated (n = 12) or with unknown vaccination status (n = 25). Of the patients with high avidity, 13 had an unknown vaccination status, one patient was registered as unvaccinated, and the remaining six patients were vaccinated, four of them with a single dose at least 23 years (range 23–38 years) before infection and two with two doses with the second one given 9 and 10 years before infection.

### Virus characterization

Overall, 137 measles virus sequences were obtained. All sequences except for the one related to an importation from Asia (MVs/Beograd.SRB/46.14, genotype D9, Fig 2) belonged to genotype D8 and 14 different sequence variants differing by 1–4 nucleotides were identified. Ten sequence variants were detected only once. In addition, there were 4 groups, each with identical sequences. These groups comprised 2, 6, 12 and 106 sequences, respectively, and the last group contained the major strain found in 9 different locations in the country. Identical sequences had been reported from other countries including Austria, Croatia, Italy and Slovenia. The main outbreak variant was detected in the country during a time period covering at least 35 weeks (epidemiological week 48 in 2014 until 30 in 2015). Sequences identical to the ones in the group of six had been found before in Germany and in B&H (Fig 2) and epidemiological information suggests a link to Germany. All other 12 sequence variants were according to BLAST analysis so far only detected in Serbia, suggesting that they evolved during the measles resurgence.

### Outbreak control measures

The public health authorities of Serbia conducted outbreak response immunization (ORI) of unvaccinated or incompletely vaccinated children aged up to 14 years in 18 districts/74 municipalities where MMR coverage for the first and/or second dose was less than 95%. Overall 10824 unvaccinated children and 8560 incompletely vaccinated children were identified. Despite ORI, MMR vaccination coverage remained <90% for both doses in 2014 and 2015 (84.0% and 85.8% for the first and 87.5% and 89.2% for the second dose) [10,11]. Patients were isolated and treated at home or in the hospital as appropriate. People who had been exposed to measles were placed under medical surveillance while children aged ≤14 years exposed within 72 hours were offered MMR vaccination. Measures proposed for the prevention of measles spread in healthcare settings were distributed to healthcare institutions. All HCWs who suffered from measles were immediately excluded from work.

## Discussion

The present report describes a thorough retrospective analysis of the measles resurgence in Serbia in 2014/15, thereby providing important information on how to counteract the future epidemics. Measles was initially introduced by and spread among citizens of B&H with temporary residence in Serbia. Most of them were students born in B&H during the war or post-war period (1992–1998) who were unvaccinated or had an unknown vaccination status. At the time of the introduction, a large measles outbreak caused mainly by the “Rostov on Don” variant of genotype D8 was ongoing in B&H [21,22]. The same virus variant was also responsible for most cases linked to the 2014/2015 measles resurgence in Serbia (77.4%, n = 106 of the 137 sequences obtained in total) and to an outbreak in Germany [23–25], where an unvaccinated Roma child from Srem (MVs/Sremska_Mitrovica.SRB/49.14, Fig 2) was probably exposed. The fact that no genotype D4 strains, responsible for the 2010/2011 outbreak [14], were found, suggests that measles virus transmission had been interrupted in Serbia after the previous epidemic.

The territorial variations in measles incidence could be related to differences in the quality of surveillance, past MMR coverage rates, presence of traveler communities and influence of vaccine objectors, especially among HCWs [26,27]. In the North of the country, mostly adults suffered from measles, probably reflecting low vaccination coverage and single dose vaccination policies in the years following vaccine introduction (e.g. 84–94% in Vojvodina between 1971–1982) [28]. The high incidence of measles among unvaccinated children ≤4 years in Southeast Serbia was related to the lowest first dose MMR coverage rates in Serbia between 2012 and 2014 recorded in the Nišava district (68.3, 70.3 and 53.8%) [11,12].

The Romani ethnic population (colloquially known as Roma), constitute one of the largest minorities in Serbia [8]. Traditionally low MMR vaccine coverage rates in Roma children resulted in recurrent oubreaks in Roma settlements [13,14]. A supplementary immunization activity (SIA) after the measles outbreak in 2010–2011 failed to vaccinate vulnerable Roma in Southeast Serbia. In 2014, MMR coverage among a representative number of Roma children was 63.3%, compared to 85.8% national coverage [29]. Insufficient awareness of vaccination benefits, vaccine safety concerns, poor access of Roma children to health care, particularly in high poverty settlements as well as poor planning of physician visits and low compliance with vaccination policies contributed to the re-emergence of measles in Roma communities in 2015. It is therefore essential to provide information about the benefits of vaccination and to address the reasons for the hesitancy with the help of Roma mediators before SIA are done [29]. The overall low success of the SIA was probably related to a lack of staff in healthcare services, poor promotion of the vaccination campaigns and inadequate cooperation of healthcare providers and local authorities [11,12].

Overall 24 measles cases below 1 year were not eligible for vaccination at the time of infection while patients aged 1–14 years were not vaccinated due to vaccine inaccessibility or parental refusal of vaccination. On average, only 79.1% (range 69.9% in 2014 to 85.9% in 2011) of all children eligible for the first dose of vaccine between 2011 and 2014 were vaccinated at the recommended age of 12–15 months, suggesting that a large number of young children remained unprotected [11,12]. A number of adult patients (63/285; 22.1%) were born before vaccine introduction in 1971.

The proportion of affected adults ≥20 years increased during the last three major measles resurgences in Serbia in 2007, 2010/11 and 2014/15 (37%, 45.1% and 67.9%, respectively)[30,31], suggesting the urgent need for conducting SIA among adults. Vaccination should not only be offered to women susceptible to rubella planning to become pregnant and people aged ≤18 years [32,33], but to susceptible adults of both genders beyond the age of 18. During the 2014/2015 measles resurgence, 88.3% of patients were adults >20 and children ≤4 years of age, who are more prone to severe measles and complications [34], resulting in a relatively high hospitalization rate.

Measles surveillance needs to be strengthened in Serbia, since many cases in the 2014/2015 resurgence were classified as endemic only because the link to importations could not be established and for several of the transmission chains, the index case was missed and countermeasures were launched too late. Another problem identified during our investigation was the lack of vaccination records in almost half of the patients (46.9%), mostly adults, making it more difficult to evaluate the contribution of non-vaccination versus vaccine failures to the measles resurgence. Further strategies should be evaluated by public health authorities regarding SIA in adults with largely unknown vaccination status. Special considerations of the vaccination algorithm of adults in relation to age and vaccination/susceptibility status should follow after careful evaluation of feasibility and cost-effectiveness. A well-functioning national vaccination information system would also strongly support effective public health countermeasures. Because of the suboptimal surveillance system and the lack of vaccination records, an IgG antibody prevalence study would be of great help to identify susceptible population groups for targeted SIA [35]. In addition, it would be helpful to capture the reasons for non-vaccination in the context of vaccination coverage surveys to develop strategies to strengthen vaccination coverage in Serbia. Since only patients who were seeking treatment were recorded, the true number of measles cases in 2014/2015 was probably underestimated.

About 10% of all notified cases in this resurgence occurred among HCWs, who infected patients, other employees and close contacts. Since measles infection of hospitalized patients poses a high risk of severe disease and fatal outcome [36,37], MMR vaccination of susceptible HCWs was recommended in the outbreak setting, but compliance was low. Mandatory MMR vaccination for all susceptible HCWs born in 1971 or later was originally scheduled for 2020, but since a substantial number of HCWs acquired measles in the current outbreak, MMR vaccination of susceptible HCWs in high-risk departments has been enabled recently [32,33]. All HCWs without documentation of vaccination with two doses of measles containing vaccine or laboratory evidence of measles or immunity are obliged to get vaccinated with two doses of MMR separated by at least 28 days. If records of only one dose of vaccine are available, an additional dose of MMR is required [32,33].

Measles IgG avidity testing in conjunction with appropriate epidemiological data is a useful diagnostic tool to detect vaccine failures [38]. Measles in a previously vaccinated patient can be classified as a primary vaccine failure (PVF) if low-avidity antibodies are detected [39]. In our study, low avidity IgG results were obtained in patients who were either unvaccinated or had an unknown vaccination status, suggesting rather primary contact with measles virus than PVF. Secondary vaccine failure (SVF), on the other hand, results in patients with high avidity measles IgG antibodies. More than one third of the patients tested had high-avidity measles IgG antibodies (n = 20, 35%), suggesting that SVF did not only occur among the six patients with documented vaccination, but probably also among patients with unknown vaccination status.

## Conclusions

Mainly adults and children ≤4 years were affected during the measles resurgence in Serbia in 2014–2015 causing a relatively high hospitalization rate. The growing number of adult patients suggests an urgent need to conduct SIA targeting all susceptible adults irrespective of age. In particular, susceptible HCWs need to be vaccinated to prevent further outbreaks in healthcare settings. Since Serbia recently experienced another large measles resurgence [40], efficient implementation of control measures including ORI is essential. The comprehensive investigation of the 2014/2015 measles resurgence and the identified problems and immunization gaps will contribute to decisions about appropriate countermeasures to stop the future measles resurgences in Serbia.

## Supporting information

S1 File Individual demographic data of measles cases registered during measles resurgence in Serbia, 2014–2015. (XLSX)
